# Particle emission rates and conditions of use for the cutting of biobased composite polyurethane foam

**DOI:** 10.12688/openreseurope.20807.3

**Published:** 2026-01-12

**Authors:** Antti Joonas Koivisto, Rossella Daniela Bengalli, Luca Ferrero, Paride Mantecca, Fabrizio Ravegnani, Letizia Verdolotti, Federica Recupido, Giuseppe Cesare Lama, Alessia Nicosia

**Affiliations:** 1 ARCHE Consulting, Wondelgem, B-9032, Belgium; 2 Institute for Atmospheric and Earth System Research (INAR/Physics), University of Helsinki, Helsinki, FI-00014, Finland; 3 POLARIS Research Centre, Dept. of Earth and Environmental Sciences, University of Milano-Bicocca, Milano, 20126, Italy; 4 CNR-ISAC, Institute of Atmospheric Sciences and Climate, National Research Council of Italy, Bologna, 40129, Italy; 5 IPCB-CNR, Institute for Polymers, Composites and Biomaterials, National Research Council of Italy, Portici, 80055, Italy

**Keywords:** Regulatory exposure assessment, REACH, Conditions of use, Exposure modelling, Risk assessment, Emission, Nanoparticle, Inhalation exposure, nano-enabled polyurethane foams, bio-based materials

## Abstract

**Background:**

Nanofillers improve polyurethane (PU) foam properties, such as thermal conductivity, mechanical properties, thermal and chemical stability, and reduce swelling. Mechanical reworking is used to shape nano-enabled PU foam material, which can result in emissions and inhalation exposure. Released fragments containing nanofillers can pose an increased risk, particularly due to inhalation exposure. This study investigates emissions from cutting bio-based composite PU panels containing functionalized silica, GasBeton ^®^, and Diatomite nanofillers, and assesses the conditions of use (CoU) for the cutting process.

**Methods:**

Concentrations were measured at the cutting site (near field; NF) and far field (FF). Process-specific concentrations were calculated for the NF and FF concentrations, and mass balance was used to calculate the cutting process emissions. The CoU assessment was conducted using the emission component with the highest risk potential. The CoU was specified as the maximum cutting rate (m ^2^/min) under reasonable worst-case (RWC) operational conditions where the NF concentration is <0.5×OEL and <1×OEL.

**Results:**

Cutting released mainly inhalable particles, with a geometric mass mean diameter of 10 µm. Aggregated average cutting emissions were 410±65 µg/min, resulting in an emission factor of 4600±730 µg/m ^2^ when using a unit density for mass concentration calculation (precautionary approach). Under RWC conditions (room volume 100 m ^3^, particle loss rate 2 1/h, NF volume 8 m ^3^, worker in NF, and air mixing flow between NF and FF 9.6 m ^3^/min), chemical-specific hazard communication is sufficient action if the cutting rate is <1.42 m ^2^/min, corresponding to 210 cut panels during an 8-hour work shift. The maximum cutting rate resulting in NF concentration <1×OEL was 2.84 m ^2^/min (420 panels).

**Conclusions:**

This study presents a method for assessing emission rates in real working conditions and quantifying broadly applicable CoU. The assessment complies with the REACH legislation criteria given for chemical safety assessment.

## Plan language summary

In REACH chemical legislation, exposure estimates must be made for all exposure scenarios where hazardous emissions occur. Exposure estimation entails estimating emissions, chemical fate and pathways, and exposure levels. Here, we quantified particle emissions from cutting cured bio-based polyurethane foam panels containing nanofillers and quantified broadly applicable conditions of use (CoU) where exposure is adequately controlled. Emissions were calculated from concentrations measured at the cutting site in near field (NF) and far field (FF) using mass balance principles. Background concentrations from ventilation air were subtracted from the measured concentrations to obtain process-specific concentrations and emission rates. Emission rates were translated to an emission factor that allows CoU assessment for the cutting in different operational conditions. Operational conditions were quantified for manual and automated cutting scenarios, where the exposures were classified as highly controlled and controlled.

## List of abbreviations

**Table:** 

Abbreviation	Term
AIHA	American Industrial Hygiene Association
*β*	Air mixing between the NF and FF
CoU	Conditions of Use
ECHA	European Chemicals Agency
ELPI	Electrical Low Pressure Impactor
FF	Far-Field
GMD	Geometric Mean Diameter
GSD	Geometric Standard Deviation
NF	Near-Field
NP	Nanoparticle
OEL	Occupational Exposure Limit
OPC	Optical Particle Counter
PM	Particulate Matter
PU	Polyurethane
PU-DIA	Polyurethane containing 2.5% diatomite powder and 2.5% functionalized silica
PU-GB	Polyurethane containing 2.5% GasBeton powder and 2.5% functionalized silica
RCR	Risk Characterization Ratio
REACH	Registration, Evaluation, Authorisation and Restriction of Chemicals
RWC	Reasonable Worst-Case
STD	Standard Deviation

## Introduction

Polyurethane (PU) is widely used in buildings, transportation, and technological applications where high-performance thermal insulation is needed. Nanofillers can improve PU properties, such as thermal conductivity, mechanical properties, thermal and chemical stability, and reduce swelling ( Arifin’ *et al.*, 2019; Fontana *et al.*, 2023; Lorusso *et al.*, 2017; Rajabimashhadi *et al.*, 2023; Verdolotti *et al.*, 2011). The fillers can be released during mechanical reworking, causing inhalation exposure risk. PU emissions have not been studied previously, but for example, thermoplastic polyurethane particle number emissions during sanding, drilling, and hand-held sawing were in the range of 3×10 ^2^ to 5×10 ^7^ 1/s, respectively ( Ding *et al.*, 2017; Koivisto *et al.*, 2025a; Neubauer *et al.*, 2017), which results in exposure levels of <5100 1/cm ^3^ ( Koivisto *et al.*, 2025a). Particle size number distributions had geometric mean diameters below 100 nm, and the released fragments were several micrometers in size, as observed in electron microscope images. Free nanoparticles were not observed. Mass emissions are not reported, although recommended exposure limit values for nanomaterials are often given as units of mass ( Mihalache *et al.*, 2017).

In registration, evaluation, authorisation and restriction of chemicals (REACH) Regulation (EC) No 1907/2006 quantitative exposure estimates need to be done for all exposure scenarios where hazardous emissions occur to support the risk characterization ( ECHA, 2016). The REACH regulation - Annex I §5.2.1 specifies that “ *The exposure estimation entails three elements: (1) emission estimation; (2) assessment of chemical fate and pathways; and (3) estimation of exposure levels*”. CoU defines the operational conditions and risk management measures that result in adequately controlled exposure (see Koivisto *et al.*, 2025b). Emission estimation is mandatory for quantifying Conditions of Use (CoU) for industrial processes ( Koivisto *et al.*, 2021; Koivisto *et al.*, 2022; Koivisto *et al.*, 2025a; Koivisto *et al.*, 2025b), which are compulsory to report in a chemical safety report ( ECHA, 2016).

Quantitative emission and exposure assessment are not usually conducted, regardless of the reporting requirements. This is because the European Chemicals Agency (ECHA) allows the use of qualitative exposure models that do not require information on emissions ( Koivisto & Jayjock, 2025). There are no particulate emissions measurement standards, such as for volatile organic compounds, e.g., the ISO 12219 series or the ASTM D5116-17 guide. Some particle emission measurement guidelines exist ( Koivisto *et al.*, 2017; Koivisto *et al.*, 2025a), but these have not been adopted in practice. Particle emission assessment is based on concentration measurements, and fundamental principles of aerosol particle dynamics are applied to calculate the emissions ( Ganser & Hewett, 2017; Hewett & Ganser, 2017; Hussein & Kulmala, 2008; Nazaroff, 1989). Particle emission models do not exist, such as for volatile substances (e.g. Keil *et al.*, 2009).

This study quantifies emissions and evaluates CoU from the cutting of bio-based composite PU panels containing functionalized silica ( Recupido *et al.*, 2023), GasBeton ^®^ ( Lama *et al.*, 2024), and Diatomite ( Recupido *et al.*, 2024). The fillers improves following properties: 1) GasBeton provides mechanical reinforcement, 2) Diatomite acts as a nucleating agent, promoting a fine and uniform cell-size distribution, which reduces thermal conductivity, and 3) The bio-based silica nanoparticles, previously functionalized to enhance compatibility with PU matrices, provide flame retardancy properties ( Recupido *et al.*, 2023). Overall, the selection of this mixture is guided by the principles of sustainability/circular economy. Specifically, GasBeton and silica nanoparticles come from wastes from industrial productions or biomass wastes (i.e. rice husk, ( Bragato *et al.*, 2024; Recupido *et al.*, 2023) whereas, diatomite is a natural filler coming from sedimentary rock of the diatoms ( Galzerano *et al.*, 2018).

The CoU assessment is based on concentration measurements in the near-field (NF) and far-field (FF), measurement of air velocities, and calculation of the emissions using an NF/FF mass-balance model ( Ganser & Hewett, 2017). An emission factor in mg per cut surface area was calculated to obtain generalized results, which were applied for CoU assessment. The CoU was specified as the maximum cutting rate (m ^2^/min) under reasonable worst-case (RWC) operational conditions, i.e., conditions that can be achieved and lead to the highest emissions, where the NF concentration is <0.5×OEL and <1×OEL. These limits correspond to the American Industrial Hygiene Association exposure categories 2 (highly controlled exposure) and 3 (controlled exposure) ( Hewett *et al.*, 2006; Torres *et al.*, 2014). This approach is considered more convenient than under REACH legislation, where exposure is considered adequately controlled if the exposure distribution 90th percentile (P90) is below the limit value, i.e., P90 < OEL ( ECHA, 2016), which allows 10% overexposure and is a holistic approach, i.e., it considers whether actions are needed without a transition in control actions ( Koivisto *et al.*, 2025b). Overall, this study demonstrates how to conduct quantitative emissions analyses using mass balance principles, how to calculate emission factors for cutting, and how to use the emission factors to quantify CoU that comply with the REACH regulation.

## Materials and methods

### Operational conditions in cutting insulation bio-based composite PU panels

Typically, PU foams consist of two Components named A and B. Component A is a mixture of bio-based polyester polyols (having Oh _n_ = 350–380 mg KOH/g and 150–160 mg KOH/g, respectively), blowing and polymerization catalysts, blowing agents, flame retardant, solid fillers, physical blowing agent (pentane), compatibilizer pentane-PU matrices, and silicone surfactants. Specifically, the polyol blend consists of 91.7% bio-based polyols and castor oil, and the concentration of additives is 8.3%. Component B is the isocyanate component (NCO content is 30.5–31.5 %). Ratio among Component A/B=100/124 (g/g), NCO/OH (mol/mol) = 1.3. The density of cured PU foams is 100±10 kg/m ^3^.

Powders of waste GasBeton ^®^ (known as autoclaved aerated concrete), diatomite, and functionalized silica are used as fillers in PU matrices. GasBeton ^®^ and diatomite were commercially available fillers (Extended Data; Koivisto *et al.*, 2025d), and functionalized silica nanoparticles were derived from rice husks as described by Bengalli *et al*. (2025). GasBeton ^®^ and diatomite were milled and sieved with a 100-mesh sieve to remove particles >150 µm prior to application. The density of the milled GasBeton ^®^ is in the range of 1.8 to 2.1 g/cm ^3,^ and the milled diatomite density is 2.3 g/cm ^3^. The total filler concentration in the PU panel is equal to 5 wt%. One set was made of 2.5 wt.% GasBeton ^®^ powder and 2.5 wt.% functionalized silica (panel named PU-GB), while the other set was 2.5 wt.% diatomite powder and 2.5 wt.% functionalized silica (panel named PU-DIA).

**Figure 1.**
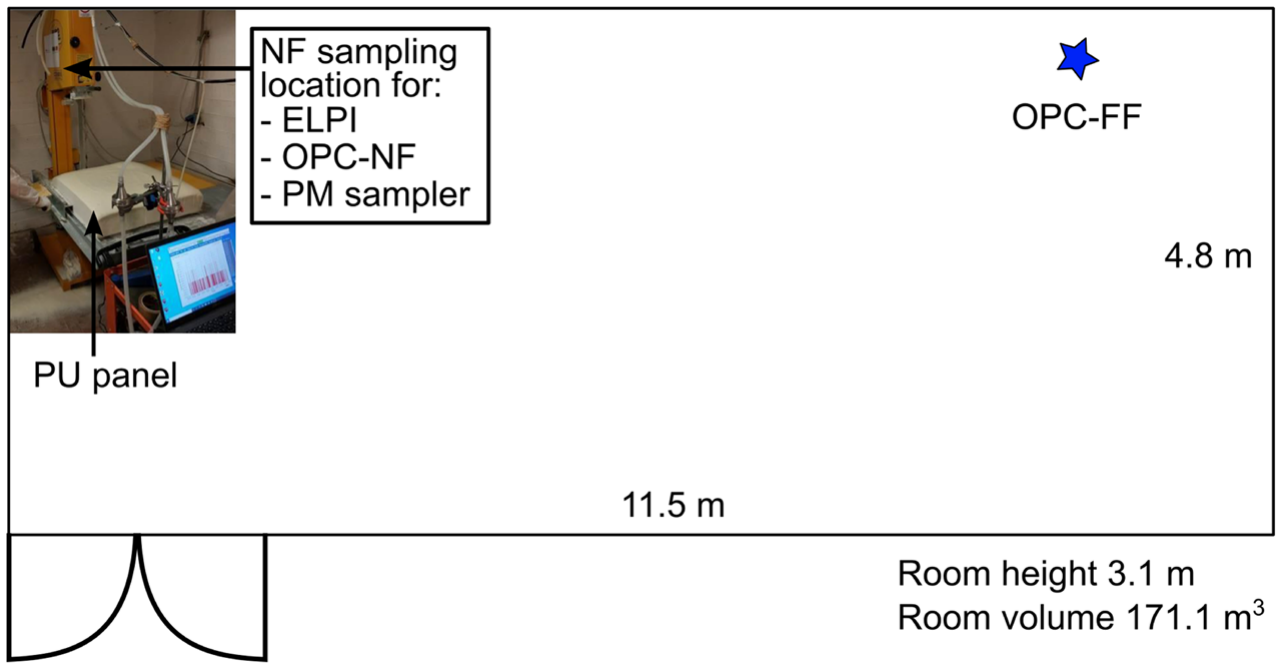
Cutting room and instruments.

PU foam panels are cut into 61×63×5 cm ^3^ panels using a band saw (EURO TSC, model CCE.650, Italy; https://www.eurotsc.com/en/products/cce-650-band-saw/). Cutting blade (Blade Widia) dimensions were 34 mm (depth) x 4120 mm (length) x 2 mm (thickness) and the blade speed was 150 m/min. The single-panel cutting duration was 9±4 min (± standard deviation; STD), which included taking the panel from a pile at 2 m from the cutting site, cutting, and placing the panel back into the pile. In total, 14 panels are cut, of which six have GasBeton filler (PU-GB) and eight have Diatomite filler (PU-DIA). The cutting room is naturally ventilated via an entrance mesh door ( Figure 1).

### Instrumentation

Particle measurements were conducted in the NF and FF by using the following online instruments:

- In the NF, particle size distributions were measured using 1) an optical particle counter (OPC-NF, Grimm mod. 11-D, Software revision: 1179-V2.03, Grimm Aerosol, Ainring, Germany) in the 0.3–30 μm size range (32 size channels) with 6 s intervals, and 2) an Electrical Low Pressure Impactor (ELPI; Dekati model ELPI+, Dekati Ltd., Tampere, Finland) in 14 size channels between 6 nm and 10 µm with 1 s intervals.
- In the FF, particle size distributions were obtained using an optical particle counter (OPC-FF, Grimm mod. 11-A, Software revision: 4-1 Rev II, Grimm Aerosol, Ainring, Germany) in the 0.3–30 μm size range (32 size channels) with 6 s intervals.

Off-line gravimetric particulate matter (PM) samples were taken in the NF by collecting the particles on absolute filters (Polytetrafluoroethylene, 1 μm porosity, Ø 47 mm) at 50 L/min flow rate (Bravo H-Plus, TCR Tecora, Italy). The mass concentrations were determined by weighing the filter before and after sample collection using an analytical balance (Mettler Toledo AX105).

The ELPI, OPC-NF, and PM sampler inlets were placed at a height of 150 cm and between the band saw blade and the operator. Sampling was performed for ELPI, OPC-FF and PM samplers using conductive sampling tubes with an inner diameter of 8 mm and a length of 205 cm for ELPI and 130 cm for OPC-FF and PM samplers. Diffusion losses by the sampling lines were not corrected because the contribution is negligible to the mass concentrations, and the loss correction cannot be applied to the PM samplers. The OPC-FF inlet was ca. 7 m from the NF site at a height of 100 cm (without sampling line).

Random airflow measurements were conducted using a single hot-wire anemometer (Testo Inc., model Testo 405i, Pennsylvania, US) with a detection limit of 0.01 m/s.

### Calculation of particle number to mass concentration

Mass concentration was derived from particle number concentration by assuming spherical particles, and the particles' effective density is constant for all particle sizes. Aerodynamic and optical diameters were assumed to be the same. As a first estimate, a unit density (1 g/cm ^3^) was used for background particles and particles released from PU-GB and PU-DIA panel cutting activities.

### Calculation of particle loss rate

Airborne particles are removed by ventilation and deposition. Gravitational settling is a primary deposition process for dust particles in the micrometer size range ( Hussein *et al.*, 2012). The particle loss rate was calculated from the concentration decay by assuming that emissions from ventilation and indoor sources are negligible, and the NF and FF concentrations are uniformly mixed ( Hussein & Kulmala, 2008).

### Aerosol dynamic model

Modellings were performed using TEAS (version 1.05 (2019), Exposure Assessment Solutions, Inc., Morgantown, MI, USA, www.easinc.co), which contains different configurations for probabilistic well-mixed room and Near-Field/Far-Field (NF/FF) models ( Ganser & Hewett, 2017; Hewett & Ganser, 2017). This study uses an NF/FF model without local exhaust ventilation for calculating NF and FF concentrations (2Box.CE.Gv; Ganser & Hewett, 2017). This is a similar model to the free software IH-MOD 2.0 provided by the American Industrial Hygiene Association ( https://ihmod.org/) or GUIDEnano ( https://www.guidenano.eu/). In the NF/FF model, all particles enter the NF air volume, where particles are dispersed to the FF air volume by air mixing between the NF and FF volumes (ß, m ^3^/min) and removed from the room via the FF air volume.

Air mixing between the NF and FF ( *β*, m ^3^/min) and an initial estimate for the emission rate ( *G*, mg/min) were calculated using the NF and FF air volumes, steady state concentrations, and the particle loss rate by general ventilation and deposition (TEAS model 2Box.CE.Gv.SS; Ganser & Hewett, 2017).


$$G=C_{OPC-FF}\times Q,\text{and}\;(1)$$

$$\beta =\frac{G}{\left(C_{OPC-NF-}C_{OPC-FF}\right)}\;(2)$$

Where *C _OPC-NF_* and *C _OPC-FF_* are measured average concentrations (µg/m ^3^) at NF and FF, respectively, and *Q* is the product of room volume (171 m ^3^) and particle loss rate (1/min). All particles are assumed to be removed from the FF volume, and the NF volume is open-faced (not in contact with walls or screens). Because the concentration measurements were carried out over the cutting period and not during the steady state period, the actual emission rate was calculated by adjusting the emission rate so that the calculated NF and FF concentrations correspond to the measured NF and FF air concentrations.

### Exposure limit values

GasBeton ^®^ consists of 70 to 90% autoclaved aerated concrete (CAS 1319-31-9), 25–35% sand, 15–30% crystalline silica (CAS 014808-60-7), and 4–9% anhydrite gypsum (CAS 7778-18-9) (Extended Data; ( Koivisto *et al.*, 2025d). PU-DIA comprises 100% diatomaceous earth (CAS 61790-53-2) (Extended Data; Koivisto *et al.*, 2025d). For risk assessment, the lowest limit values were used for GasBeton ^®^ as the respirable fraction and diatomite as the inhalable fraction ( Table 1). The recommended exposure limit value of 0.3 mg/m ^3^ was applied for functionalized silica, given as an 8-h TWA for the respirable fraction ( Mihalache *et al.*, 2017). The limit value of 4 mg/m ^3^ given as an 8-h TWA for organic dust was applied for PU ( IFA, 2016).

**Table 1.** Compositions and OELs for GasBeton ^®^ and Diatomite.

Substance (CAS no.)	Concentration	Respirable, mg/m ^3^	Inhalable, mg/m ^3^
GasBeton®			
Autoclaved aerated concrete (CAS 1319-31-9)	70 to 90%	2.5 to 5 as inert dust ( IMA, 2024)	4 to 10 as inert dust ( IMA, 2024)
Sand (CAS N/A)	25–35%	2.5 to 5 as inert dust ( IMA, 2024)	4 to 10 as inert dust ( IMA, 2024)
Crystalline silica (CAS 014808-60-7)	15–30%	0.05 to 0.15 ( IMA, 2024)	Not considered here
Anhydrite gypsum (CAS 7778-18-9)	4–9%	1.5 to 6 ( IFA, 2016)	4 to 15 ( IFA, 2016)
Diatomite			
Diatomaceous earth (CAS 61790-53-2)	100%	Not specified	1 to 5 ( IMA, 2024)

## Results

**Figure 2.**
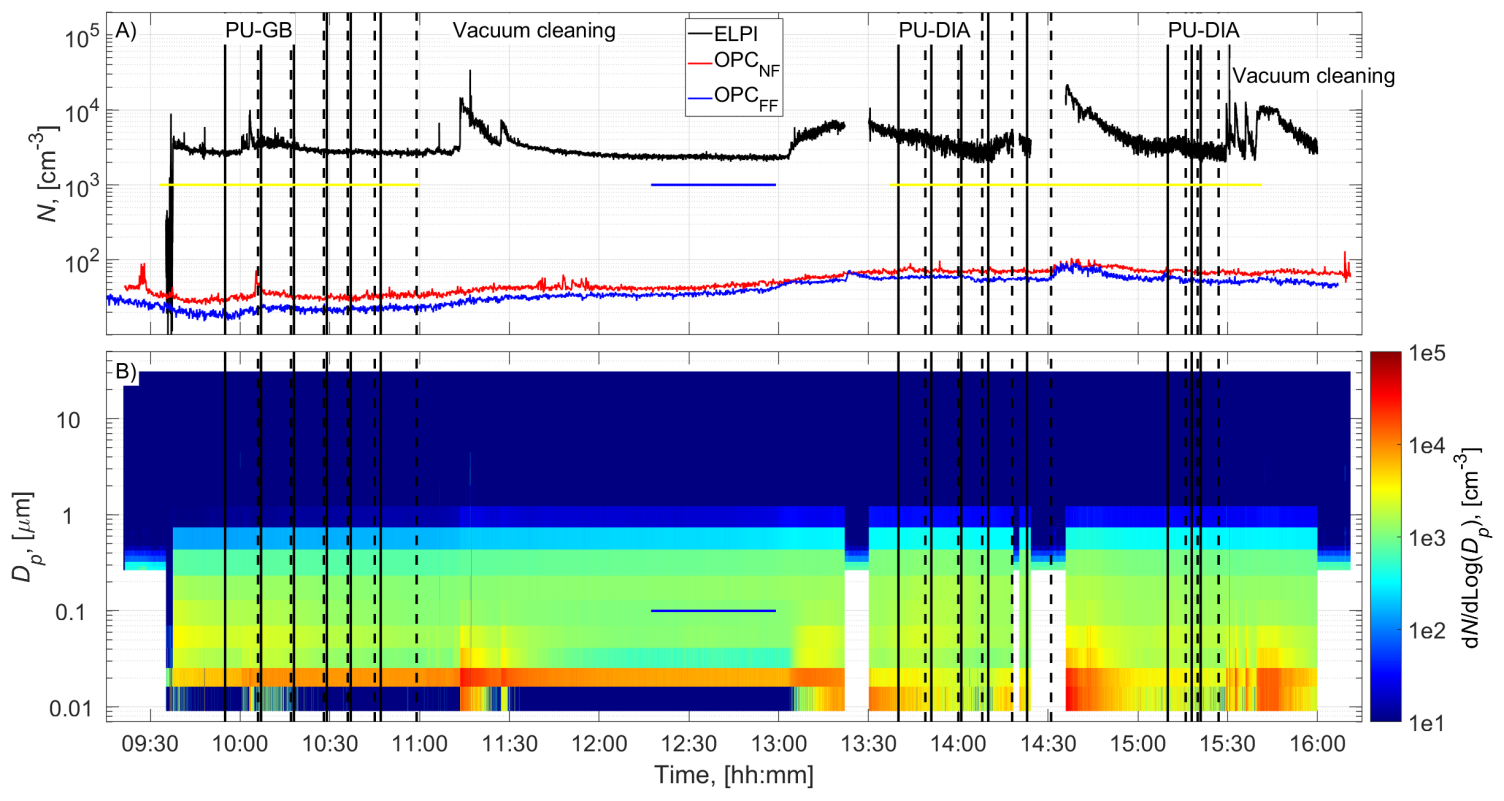
Particle number concentration time series: **A**) total concentrations measured in the NF and FF, and **B**) size distributions measured in the NF using ELPI and OPC-NF. The vertical solid and dashed black lines show the start and end times of the cutting for each panel. The blue and yellow horizontal lines show the background concentration measurement period and gravimetric sampling periods, respectively.

**Figure 3.**
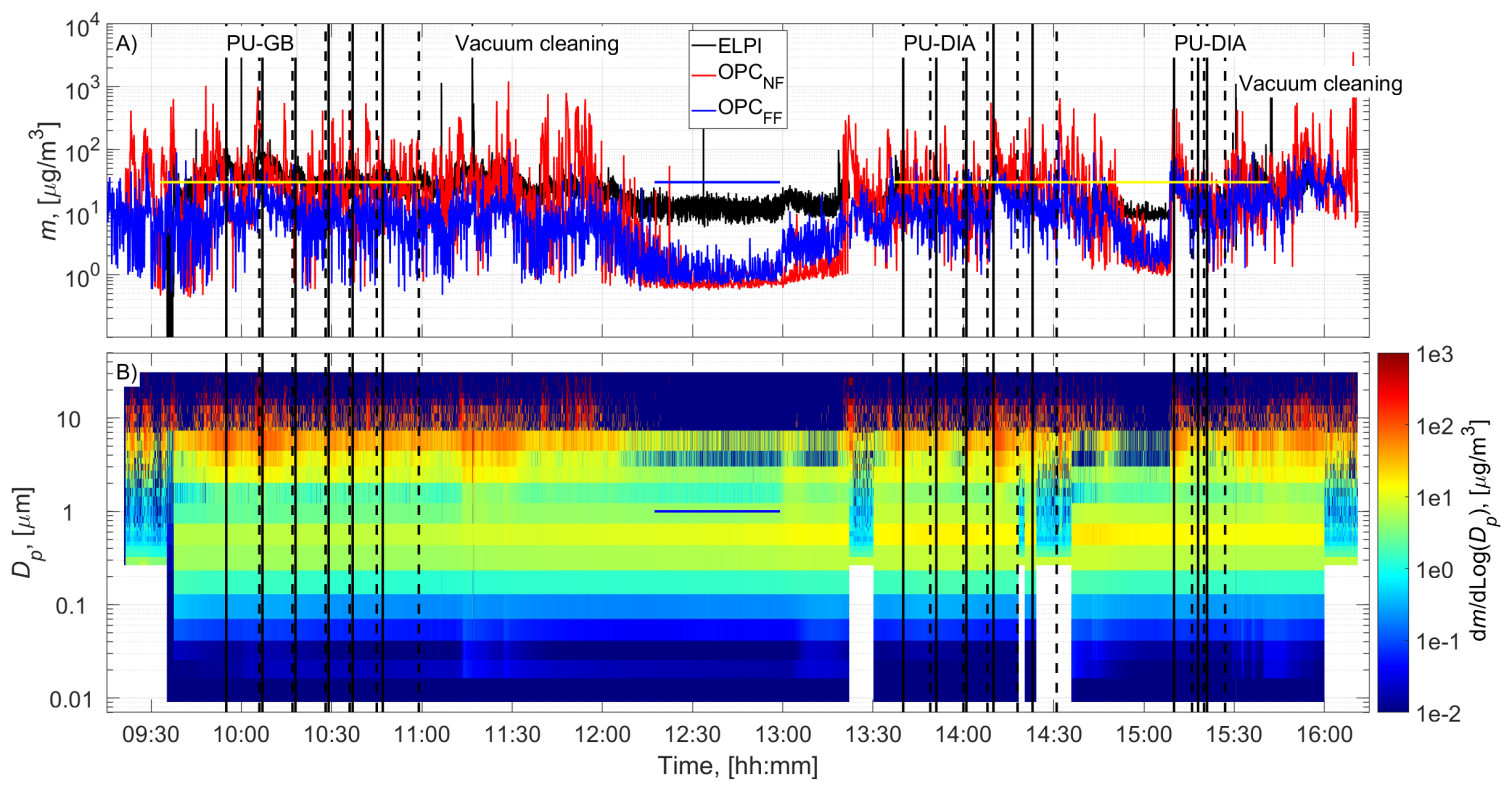
Particle mass concentration time series: **A**) total concentrations measured in the NF and FF, and **B**) size distributions measured in the NF using ELPI and OPC-NF. The vertical solid and dashed black lines show the start and end times of the cutting for each panel. The blue and yellow horizontal lines show the background concentration measurement period and gravimetric sampling periods, respectively.

The cutting room temperature was 22±1˚C (±STD). The random airflow velocity at the mesh door was 0.17±0.08 m/s (±STD), and at the NF without an operator, it was 0.022±0.03 m/s (±STD). PU-GB panels were cut from 9:55 to 10:59, and PU-DIA panels were cut from 13:40 to 14:31 and from 15:10 to 15:27 ( Figure 2 and Figure 3). Particle number concentration and mass concentration time series are shown in Figure 2 and Figure 3, respectively.

### Background concentration

Before cutting, panels were moved to the room, instruments were set up, and residual dusts from the previous cutting operations were wiped from the band saw, increasing the NF concentrations ( Figure 2 and Figure 3). This was not considered to represent background concentration without processes; therefore, the background concentration was based solely on concentrations measured during a lunch break ( Figure 2 and Figure 3). A similar background level was achieved between 15:00 and 15:10, indicating that the background concentration is representative of the entire work shift. Background concentrations were specified separately for each instrument (ELPI, OPC-NF, and OPC-FF; Table 2; Figures ED1 and ED2, Extended Data; ( Koivisto *et al.*, 2025d)). The background concentration ratios between the NF and FF OPCs were 1.3 and 0.9 for particle number and mass concentrations ( Table 1), respectively, and the background size distributions were within a factor of ~2 similar in the NF and FF (Figures ED1 and ED2, Extended Data; ( Koivisto *et al.*, 2025d)). This indicates that the background levels were similar at NF and FF locations.

**Table 2.** Background and cutting particle concentrations. Abbreviations: *N*/ *m*, number/mass concentration; GMD, geometric mean diameter; GSD, geometric standard deviation of the GMD. Averaging times: Background 12:17-12:59, PU-GB and PU-DIA 9:55-10:59, 13:40-14:31, and 15:10-15:27, PU-GB 9:55-10:59, and PU-DIA 13:40-14:31, and 15:10-15:27.

Scenario	Units	ELPI			OPC-NF			OPC-FF		
		*N*/ *m*	GMD, µm	GSD	*N*/ *m*	GMD, µm	GSD	*N*/ *m*	GMD, µm	GSD
Process concentrations (With background)										
Background	#/cm ^3^	2720	0.038	2.8	46	0.29	1.1	37	0.32	1.2
PU-GB and PU-DIA	#/cm ^3^	3330	0.034	2.8	55	0.30	1.3	40	0.33	1.3
PU-GB	#/cm ^3^	3390	0.033	2.5	35	0.31	1.4	22	0.34	1.4
PU-DIA	#/cm ^3^	3270	0.035	3.2	74	0.23	1.3	56	0.33	1.3
Background	µg/m ^3^	15	2.0	4.1	1.1	1.0	5.1	1.2	0.77	2.7
PU-GB and PU-DIA	µg/m ^3^	32	3.1	3.0	41	9.5	2.3	12	5.1	2.5
PU-GB	µg/m ^3^	42	4.1	2.5	49	10.1	2.2	11	5.5	2.3
PU-DIA	µg/m ^3^	23	1.8	3.2	34	8.7	2.4	14	4.9	2.7
Process concentrations (background subtracted)										
PU-GB and PU-DIA	#/cm ^3^	890	0.020	2.3	8.6	0.37	1.8	2.9	0.49	2.0
PU-GB	#/cm ^3^	750	0.025	1.8	1.8	0.98	2.4	0.4	1.7	2.1
PU-DIA	#/cm ^3^	1390	0.019	2.8	28	0.31	1.4	19	0.34	1.4
PU-GB and PU-DIA	µg/m ^3^	18	4.4	1.6	40	10.1	2.0	11	6.36	1.9
PU-GB	µg/m ^3^	37	5.4	1.5	48	10.6	2.0	10	6.36	1.8
PU-DIA	µg/m ^3^	15	3.9	1.5	33	9.8	1.9	12	6.35	1.9

### Process concentrations

Process particle number concentrations for PU-GB and PU-DIA were on average 1.3, 1.2, and 1.1 times higher than the background number concentration measured by the ELPI, OPC-NF, and OPC-FF, respectively ( Table 2). The number concentration increase was mainly associated with particles <100 nm for ELPI measurements and particles <1 µm for OPC measurements ( Figure 2 and Figures ED1 and ED3; Extended Data; ( Koivisto *et al.*, 2025d). The highest relative number concentration increase was associated with particles >1 µm for all instruments ( Figure 2 and Figures ED1, ED3, and ED5; Extended Data; Koivisto *et al.*, 2025d).

Process particle mass concentrations for PU-GB and PU-DIA were on average 2.2, 37.8, and 10.0 times higher than the background mass concentration measured by the ELPI, OPC-NF, and OPC-FF, respectively ( Table 2). The mass concentration increase was mainly associated with particles >1 µm for all instruments ( Figure 3 and Figures ED2 and ED4; Extended Data; Koivisto *et al.*, 2025d). The upper detection limit for ELPI is 10 µm, and it did not capture most of the released particles' mass ( Figure 3 and Figure ED6, Extended Data; Koivisto *et al.*, 2025d). Based on geometric mass mean diameter (Table 2), the released particles were mainly inhalable particles ( Schwarz *et al.*, 2018). Background particle density is expected to differ from process particle density, and the uncertainty in this difference is not known because the sampling periods, averaging times, collection/ measurement size ranges, and detection techniques differ.

The gravimetric analysis revealed NF mass concentrations of 25.2 and 35.3 µg/m ^3^ during the PU-GB (period 9:33 – 11:00) and PU-DIA (period 13:37 – 15:41) cutting activities, respectively. This results in a weighted average concentration of 31.1 µg/m ^3^ during the 211 min total sampling time. The gravimetric mass concentrations for PU-GB were 0.68 and 0.53 times the respective mass concentrations calculated from the ELPI and OPC-NF, and for PU-DIA, 2.43 and 1.08 times. The weighted average mass concentration was 1.77 and 0.78 times the mass concentrations calculated from the ELPI and OPC-NF.

The concentrations are expected to be similar between PU-GB and PU-DIA cutting because the matrix and filler weight concentrations are the same. Thus, the unit density used for mass calculations should result in a systematic error, which was not seen here. The difference was likely caused by emissions unrelated to cutting, as the PU-GB gravimetric sample contained emissions from pre-activities, and the PU-DIA gravimetric sample included emissions from cleaning ( Figure 2). Other uncertainties are related to the differences between different detection techniques, size ranges, and sampling conditions.

### Particle loss rates

Coarse particles may settle before reaching the FF measurement location. To obtain a representative loss rate, loss rates were calculated from both NF and FF concentrations to determine if there were any significant differences. When no activities were in the room, particle loss rates were calculated from OPC-NF and OPC-FF mass concentrations. The background mass emission rate was assumed to be insignificant compared to the mass loss rate. The fitting functions and parameters are presented in Figures ED7-ED10, Extended Data ( Koivisto *et al.*, 2025d). The loss rates were at NF 15.9 and 13.2 1/h, and at FF 8.9 and 13.9 1/h. The average loss rate was calculated using NF and FF loss rates, which resulted in 13.0±2.6 1/h (± STD). The average loss rate was applied for the calculations because the conditions for the loss rate should have remained the same for both NF and FF. A loss rate assessment based on two measurements in NF and FF, respectively, is insufficient for uncertainty assessment.

### Emission rates and air mixing

Because of incomplete air mixing, the emission rates must be calculated using the NF/FF model. The standard NF/FF model (2Box.CE.Gv) under steady state can solve the emission rate and the *β* (2Box.CE.Gv.SS; Ganser & Hewett, 2017). The OPC measurements obtained NF and FF concentrations, and the particle loss rates were obtained from the mass concentration decay fittings (Figures ED7–ED10, Extended Data; Koivisto *et al.*, 2025d). Continuous emissions were assumed because the emission calculation used the average concentrations. Particle emissions originate from the cutting and resuspension of settled dust, which cannot be distinguished here. The calculations were performed using an NF volume of 8 m ^3^, a room volume of 171 m ^3^, and the task duration and emission time corresponded to the cutting start and end times.

The process concentrations were calculated for a period with an increasing concentration phase when the cutting activity was started. Thus, the emission rate solved using the 2Box.CE.Gv.SS model is an initial estimate of the emission rate (see an example of the calculation in Extended Data, Section 4, “The initial estimation of the emission rate and beta”; Koivisto *et al.*, 2025d), which was adjusted so that the calculated concentrations correspond with the OPC-NF and OPC-FF mass concentrations (see an example of the calculation in Extended Data, Section 5, “Emission rate correction”; Koivisto *et al.*, 2025d). Calculation of the *β* depends only on the relative difference between the NF and FF concentrations. It was assumed that concentration increases and decreases similarly in the NF and FF, and no adjustments were made to *β*, which would require time- and spatial-resolved calculations.

Emission rates and *β* were calculated for the aggregated cutting of PU-GB and PU-DIA panels and separately for PU-GB and PU-DIA panels ( Table 3). The *β* increases as the particle loss rate increases because the more particles are lost from the FF, the more particles need to be fed into the FF to obtain the OPC-FF concentration ( Table 3). Measured random air velocity was 0.022±0.03 m/s without activity, corresponding to an average *β* value of 15.8 m ^3^/min for an open-faced cube with side length 2 m. This is in the range of calculated *β* values ( Table 3).

**Table 3a.** Cutting scenario variables and calculated parameters. Modelling parameters: room volume 171 m ^3^, near field volume 8 m ^3^, continuous emission during cutting, loss rate mean-STD, mean, and mean+STD, air mixing between NF and FF ( *β*, m ^3^/min) calculated using ^a^2Box.CE.Gv.SS, ( Equation 2), and emission rate (G, µg/min) was adjusted so that calculated *C _NF_* and *C _FF_* concentrations were like OPC-NF and OPC-FF, respectively, using ^b^2Box.CE.Gv.

Scenario				Calculated parameter	Loss rate, 1/h		
Panel (cut period)	OPC-NF, µg/m ^3^	OPC-FF, µg/m ^3^	Emission time, min		10.4	13.0	15.5
PU-GB+PU-DIA (9:55-10:59, 13:40-14:31 and 15:10-15:27)	39.7	10.8	140	*G*, µg/min	405	333	493
				*β*, m ^3^/min ^a^	13.8	11.4	17.0
				*C _NF_*, µg/m ^3^ ^b^	39.6	39.7	39.7
				*C _FF_*, µg/m ^3^ ^b^	10.1	10.7	10.8
PU-GB (9:55-10:59)	47.9	10.0	64	*G*, µg/min	383	308	455
				*β*, m ^3^/min ^a^	9.8	7.8	11.7
				*C _NF_*, µg/m ^3^ ^b^	47.9	48.0	47.9
				*C _FF_*, µg/m ^3^ ^b^	9.5	9.3	9.6
PU-DIA (13:40-14:31 and 15:10-15:27)	32.7	12.2	76	*G*, µg/min	457	369	545
				*β*, m ^3^/min ^a^	21.4	17.2	25.7
				*C _NF_*, µg/m ^3^ ^b^	32.7	32.7	32.7
				*C _FF_*, µg/m ^3^ ^b^	11.5	11.4	11.7

**Table 3b.** Model parametrization. All distributions are described as uniform.

Parameter	Value	Comment
Emission rates	PU-GB: 322 to 442 µg/min PU-DIA: 385 to 529 µg/min	The calculation is based on OPC-NF and OPC-FF process-specific mass concentrations, assuming unit density. The range is specified by the mean emission rate ± STD.
Emission times	PU-GB: 0 to 59 min PU-DIA 1 ^st^ cut: 225 to 276 min PU-DIA 2 ^nd^ cut: 315 to 332 min	Start time 9:55 set to 0 min. End time corresponds to the gravimetric sampling end time for PU-DIA cutting.
Room volume	154 to 171 m ^3^	An empty room volume. Room loading was <10 % of the total room volume. Overestimation of room volume overestimates the ventilation air volume flow (dilution overestimated)
Particle loss rate by ventilation and deposition	10.4 to 15.5 1/h	Based on NF and FF concentration decay average ± STD.
NF volume	4 to 12 m ^3^	Average 8 m ^3^ with a range of ±4 m ^3^. This represents a cube with a side length ranging from 1.6 to 2.3 m, which mimics the operator working distance for cutting (0.8 m) and replacing the panel with a new one (1.2 m).
Air mixing between NF and FF ( *β*)	PU-GB: 7.8 to 11.7 m ^3^/min PU-DIA: 17.2 to 25.7 m ^3^/min	The range of loss rate is adopted from Table 3a.

Increased loss rates increase the emissions required to get the OPC-NF and OPC-FF concentrations ( Table 3). However, emissions are expected to be independent of ventilation rate, and emission variation is associated with uncertainties related to the concentration measurements used to calculate loss rates and *β*. Cutting emissions were on average 382±60 and 457±72 µg/min (± STD) for PU-GB and PU-DIA panels, respectively. Average aggregated emissions for PU-GB and PU-DIA panels were 410±65 µg/min (± STD), which is within the one standard deviation variation of individual panel cutting emission ranges. The calculated NF and FF concentrations were 100.0% to 100.1% and 92.6% to 100.1% of the OPC-NF and OPC-FF concentrations, respectively, showing reasonable agreement with the measurements ( Table 2).

Calculated emissions represent the total activity emissions, including resuspension of particles. An emission factor was calculated as µg per m ^2^ of cut surface area, which can be used to generalize the results for different cutting conditions. All emissions are assumed to originate from cutting, and cutting velocity does not affect the total mass emitted. Emission rate was multiplied by emission time ( Table 3) and divided by the surface area of the cut panel (0.893 m ^2^). Total emissions were calculated as aggregated emissions for PU-GB and PU-DIA panels (410±65 µg/min), resulting in 57400±9200 µg during 140 min of cutting 14 panels. This results in an aggregated emission factor of 4600±730 µg/m ^2^ (±STD).

The cutting surface area is slightly higher because the initial PU blocks were ca. 5% larger than the cut panel, which would reduce the emission factor by a factor of 1.1. However, because exact values were not measured, this is not considered here (precautionary approach). The emission factor was calculated using unit density, which resulted in 1.28 times higher OPC-NF mass concentrations than the weighted average gravimetric concentration. Because number-to-mass conversion is linearly proportional to density, the calculated emissions can be corrected by multiplying by a factor of 0.78. This results in an aggregated emission rate of 320±51 µg/min and an emission factor of 3586±572 µg/m ^2^ (particle density 0.78 g/cm ^3^).

### Comparison of calculated NF concentrations with measurements

Concentrations in the cutting scenario were reproduced using the 2Box.CE.Gv model using the parameters in Table 3. The simulation report for NF concentrations is given in Extended Data, Section 6, “Recalculation of the cutting scenario” ( Koivisto *et al.*, 2025d).

The gravimetric measurement times were normalised to correspond to the simulation time by a factor of 0.64, corresponding to the PU-GB and PU-DIA total sampling time of 211 min divided by the simulation time of 332 min. This resulted in a weighted average NF concentration of 19.8 µg/m ^3^ while the simulated NF concentration was 23.4±2.6 µg/m ^3^ on average (± STD). The simulated concentrations overestimated the measured concentrations by a factor of 1.18. The difference is mainly associated with the unit density assumption and underlying assumptions in the same sampling efficiencies, dispersion of pollutants, and analytical methods. If a density of 0.78 g/cm ^3^ is used instead of unit density, the simulated NF concentration is underestimated by a factor of 0.92. The average FF concentration was 4.9±0.5 µg/m ^3^ (simulation report not shown), indicating an underestimation compared to the OPC-FF concentration of 11 µg/m ^3^ by a factor of 2.2.

### Conditions of use

The PU matrix, with an OEL of 5 mg/m ^3^, is the limiting factor in CoU assessment based on the PU-GB and PU-DIA panels' compositions, maximum filler concentrations in the panels, and exposure limit values ( Table 1). For PU-GB, the second relevant component is crystalline silica, which causes a 21% lower risk than the PU matrix. The functionalized silica is the second key component for PU-DIA, resulting in a 67% lower risk compared to the PU matrix.

CoU was assessed using a 100 m ^3^ room (free space), particle loss rate by ventilation 2 1/h (particle deposition ignored), NF volume of 8 m ^3^, the worker is in NF, and air mixing flow between NF and FF 9.6 m ^3^/min, representing average *β*-STD ( Table 3), and average emission factor of 4600 µg/m ^2^. This is assumed to represent worst-case operational (RWC) conditions for continuous panel cutting over an 8-h work shift. Cutting rate was selected so that PU risk characterization ratio (RCR) is <0.5×OEL, i.e., PU-concentration/PU-OEL<0.5. This corresponds to the American Industrial Hygiene Association (AIHA) exposure category 2 (highly controlled exposure), and chemical-specific hazard communication is required action ( Hewett *et al.*, 2006; Torres *et al.*, 2014).

A 6.5 mg/min emission rate complies with this condition, resulting in an NF concentration of 2.498 mg/m ^3^ (including fillers) (Extended Data, Section 7, “CoU assessment”, Koivisto *et al.*, 2025d). The cutting rate during measurements was 0.089 m ^2^/min (14 panels cut in 140 minutes), which resulted in an emission rate of 410±65 µg/min. This corresponds to a 15-times higher cutting rate of 1.42 m ^2^/min or 210 cut panels during 8 hours. This represents a cutting speed 4.4 times higher than in the measured scenario.

If the cutting rate is doubled to 2.84 m ^2^/min (420 panels) during an 8-h work shift, the NF exposure is 4.995 mg/m ^3^ (including fillers). The exposure is considered controlled (AIHA exposure category 3), but in addition to chemical-specific hazard communication, exposure surveillance, medical surveillance, and work practice evaluation are also necessary ( Hewett *et al.*, 2006; Torres *et al.*, 2014). This scenario represents a more automated cutting scenario featuring a sawdust collector to maintain the system's operation.

### Limitations and recommendations

Room ventilation was conducted naturally via a mesh door approximately 3 m from the cutting site. Thus, the FF measurements may underestimate the concentrations removed from the room via ventilation. If all particles are assumed to be lost thorough ventilation, the calculated emission rate is directly proportional to the FF concentration ( Ganser & Hewett, 2017). This can underestimate the emissions calculated from the OPC-NF/FF measurements. Therefore, the 4600±730 µg/m ^2^ emission factor calculated using unit density is recommended until the model can be independently validated under well-controlled conditions.

Generally, the released fragments show nanofillers mainly embedded in or protruding from the matrix, and toxicity modulation by nanomaterials is shown to be limited or not detectable ( Wohlleben *et al.*, 2024). Here, electron microscopy analysis was not performed to evaluate the fraction of free nanofillers embedded in or protruding from the matrix. Following the precautionary principles, all nanofillers were assumed to be fully bioavailable. Assessing the fraction of fillers that can become bioavailable can improve risk assessment related to nanofillers.

In the CoU assessment, emissions were assumed to be linearly scalable, which has not been confirmed. The ventilation rate could not be determined, and the calculated loss rate was a combination of particle removal by ventilation and deposition. Resuspension emissions are related to the working activity and can increase or reduce emissions. In emission scaling, particle resuspension is expected to become a more significant source than in the measured scenario because of higher dust accumulation. The CoU assessment was conducted using a loss rate of 2 1/h, representing particle removal by ventilation. The CoU complies if the particle loss rate by deposition is higher than the particle resuspension rate during an 8-h period. The use of a sawdust collector is recommended.

Currently, the REACH regulation does not require specific surface area or particle number concentration measurements, but if legislation changes, these emission units should also be considered.

Unknown uncertainties related to this study included differences related to the instruments’ sampling and analytical methods, density not being constant with particle diameter, air mixing, ventilation rate, deposition rate, resuspension rate, fillers' bioavailable fraction, representativeness of FF measurements for particle losses via ventilation, and cutting surface area, and cutting emissions being linearly dependent on the cutting surface area. Particle loss by FF concentrations is not a precautionary assumption. Still, the remaining unknown uncertainties do not have a significant impact on the emission rate or are followed by precautionary assumptions. The overall uncertainty is considered adequately specified.

It is known that silica nanoparticles (NPs) could exert toxic effects after inhalation in in vitro lung cells or in vivo studies. The toxicity or inflammatory potential of silica NPs depends on their synthesis process, shape, size, crystallinity, and on the cell type and time of exposure to NPs ( Inoue *et al.*, 2021; Lehman *et al.*, 2016). Indeed, bio-based silica NPs derived from rice husks, either functionalized or not with polyols, have been proven to be safe, in terms of cytotoxicity, oxidative stress and inflammatory potential, in alveolar lung cells (A549), monocytes (THP-1) and in a co-culture model of the alveolar epithelium in comparison to commercial pyrogenic silica NPs, ( Bengalli *et al.*, 2025). This evidence suggests that the use of bio-nanofillers seems to be a safer strategy as an alternative to conventional nanomaterials used in the production of PUR foams.

## Conclusions

Under REACH legislation, exposure estimation in chemical safety reports involves assessing emissions, chemical fate, and exposure. Emission assessments are rarely conducted because exposure assessments are often performed using qualitative exposure models that do not require information on emissions. In this study, we conducted a chemical safety assessment that complied with the REACH legislation criteria for exposure estimation. The safety assessment was conducted for bio-based composite PU foam panel cutting containing PU-GB or PU-DIA fillers and functionalized silica nanoparticles. 

NF and FF particle concentration measurements were used to calculate 1) particle loss rates by ventilation and deposition, 2) air mixing between the NF and FF, and 3) emission rates. Particle loss rates were calculated by fitting a first-order decay function to the concentrations measured in the absence of other sources, except for particles transported by incoming ventilation air. Emissions and air mixing between the NF and FF were calculated using the standard NF/FF model. The NF-FF flow rate calculated using the measured air flows resulted in a value similar to the estimated value from NF/FF measurements. The OPC-NF mass concentrations calculated using unit density were 1.28 times higher than the weighted average concentration measured gravimetrically. The emission rates (average 410±65 µg/min) calculated using the unit density resulted in 1.18 times higher simulated NF concentrations than those measured NF concentrations using gravimetric methods. In contrast, the corrected density resulted in underestimation by a factor of 0.92. Following precautionary principles, the CoU assessment was performed using the emission factor based on the unit density (4600±730 µg/m ^2^).

CoU was assessed by defining RWC operational conditions and adjusting the cutting rate (m ^2^/min) so that RCR was <0.5 and <1 times OEL for the component causing the highest risk, which was the PU matrix for both panel types. This resulted in a cutting rate of 1.42 and 2.84 m ^2^/min, corresponding to the cutting of 210 and 420 panels during an 8-hour work shift. This corresponds to 15 and 30 times higher cutting rates than in the measured scenario. A sawdust collector is recommended to minimize exposure to resuspension particles. Resuspension emissions were considered only for the measured scenario activity, which increases as the cutting rate increases.

## Ethic and consent

Ethical approval and consent were not required.

## Institutional review board statement

Not applicable.

## Informed consent statement

Not applicable.

## Data Availability

Zenodo: Extended data for “Particle emission rates and conditions of use for the cutting of biobased composite polyurethane foam”. https://doi.org/10.5281/zenodo.15727267 ( Koivisto *et al.*, 2025d). The project contains the following underlying data: Extended data.docx: Concentration plots, mass loss rate fittings, and TEAS simulation reports Material safety data sheet for Gasbeton ^®^ Material safety data sheet for Diatomite Data are available under the terms of the Creative Commons Attribution 4.0 International license (CC-BY 4.0) ( https://creativecommons.org/licenses/by/4.0/).
