# Supplementary material for: Particle emission rates and conditions of use for the cutting of biobased composite polyurethane foam
Source: Open Res Eur. 2026 Jan 12;5:207. Originally published 2025 Jul 30. [Version 3] doi: 10.12688/openreseurope.20807.3 (PMC12800609; doi:10.12688/openreseurope.20807.3)
Supplement: Peer review history

# Reviewer report: Reviewer response for version 3

**DOI:** 10.21956/openreseurope.24427.r67997

**Published:** 2026-01-19

**Authors:** Yuji Fujitani

**Affiliations:** 1 National Institute for Environmental Studies, Tsukuba, Japan

Page 9 right column, first paragraph

 The additional explanation about using an average loss rate is helpful. However, the original request was to explicitly report the loss rates for each period. Including the period-specific values (e.g., 15.9 and 13.2 h⁻¹ at NF for lunch and afternoon) would improve clarity.

 Apart from this point, the reviewer has no further comments. The reviewer thanks the authors for their efforts in revising the manuscript.

Is the study design appropriate and does the work have academic merit?

Yes

Is the work clearly and accurately presented and does it cite the current literature?

Partly

If applicable, is the statistical analysis and its interpretation appropriate?

Not applicable

Are all the source data underlying the results available to ensure full reproducibility?

No

Are the conclusions drawn adequately supported by the results?

Yes

Are sufficient details of methods and analysis provided to allow replication by others?

Partly

Reviewer Expertise:

My expertise is in aerosol measurement and inhalation exposure assessment. I can evaluate the experimental design, concentration measurements, and emission/exposure assessment.

I confirm that I have read this submission and believe that I have an appropriate level of expertise to confirm that it is of an acceptable scientific standard.

# Reviewer report: Reviewer response for version 3

**DOI:** 10.21956/openreseurope.24427.r67996

**Published:** 2026-01-13

**Authors:** Ramazan Şener

**Affiliations:** 1 Bandırma Onyedi Eylül University, Balıkesir, Turkey

The manuscript can be accepted in its current form for publication.

Is the study design appropriate and does the work have academic merit?

Yes

Is the work clearly and accurately presented and does it cite the current literature?

Partly

If applicable, is the statistical analysis and its interpretation appropriate?

Not applicable

Are all the source data underlying the results available to ensure full reproducibility?

Partly

Are the conclusions drawn adequately supported by the results?

Partly

Are sufficient details of methods and analysis provided to allow replication by others?

Partly

Reviewer Expertise:

Computational fluid dynamics; Particle emissions; combustion

I confirm that I have read this submission and believe that I have an appropriate level of expertise to confirm that it is of an acceptable scientific standard.

# Reviewer report: Reviewer response for version 2

**DOI:** 10.21956/openreseurope.23006.r63243

**Published:** 2026-01-05

**Authors:** Samet Uslu

**Affiliations:** 1 Karabuk University, Karabuk, Turkey

This article offers a valuable contribution in terms of quantifying particulate emissions released during the cutting of bio-based PU foam panels containing nano-fillers and deriving REACH-compliant "Conditions of Use (CoU)" from these emissions. The NF/FF measurements and the two-box (2-box) mass balance approach are robust due to their applicability in practical workplace conditions. In particular, the goal of generalizing the emission rate to different plant/operation conditions by converting it to an "emission factor (µg/m²)" is a sound approach.

 However, the study is currently lacking in terms of reproducibility and the quantitative/transparent presentation of uncertainties; furthermore, the reliability of the results is limited if some critical assumptions on which the CoU results are based (especially density, FF representing ventilation loss, resuspension contribution, and "linear scalability") are not more clearly justified.

 1- In the introduction, the motivation for choosing functionalized silica + GasBeton® / diatomite should be summarized briefly but technically: (i) industrial prevalence/recycling (waste GasBeton®), (ii) effects of functionalized silica on PU morphology/thermal/fire etc., (iii) risk perspective (crystalline silica content etc.).

 Suggestion: Add a 4-6 sentence “material selection rationale” to the last paragraph of the introduction and link the filler properties (at least size distribution, specific surface, presence/absence of crystalline silica) to the Extended Data.

 2- The text provides the cutting time (9±4 min per panel), but parameters that can significantly affect emission, such as cutting speed, blade type/tooth spacing, belt speed(s), pressure force/operator behavior, and moisture content, are not sufficiently detailed. There is also an emphasis on "natural ventilation mesh door," but it remains unclear whether FF represents "room removal" since ventilation flow rate/ACH is not measured; the authors also state that this could affect the emission factor.

 3- Mass concentrations, when converting from count to mass:

 -it is assumed that the particles are spherical,

 -that the effective density is independent of size,

 -that the aero/optical diameters are the same;

 1 g/cm³ is taken as the starting value.

 Then, gravimetric comparison shows that this approach can change the emission factor (with a correction of 0.78 g/cm³, the emission factor drops to ~3586 µg/m²). This section is currently a bit confusing for the reader.

 Suggestion:

 A small box/subheading in the main text: “Mass conversion sensitivity: density & diameter assumptions”

 The sensitivity of CoU results with at least 2 scenarios: (i) 1 g/cm³ (cautious), (ii) gravimetrically compatible effective density. How the CoU “cutting rate limit” values ​​change in these two scenarios can be presented in a table. Discuss the effect of the “aerodynamic = optical” assumption (OPC’s refractive index dependence, etc.).

 4- The text reports the loss rate as two values ​​from NF and FF; it is not clear why there are two values ​​and which periods they correspond to without going to the Extended Data.

 Also, there is the expression “0.022±0.03 m/s → β=15.8 m³/min (2 m³ open surface)” for β; but it cannot be reproduced because the formula/geometric relationship is not shown.

 Suggestion:

 Clearly state the loss rates with period labels such as “lunch” and “afternoon” (like the format suggested by the reviewer).

 2–3 lines of calculation for β conversion:

 β=U×A (using effective area/“open-faced cube” definition).

 Add G and β definitions to Table 3a heading (G=emission rate; β=NF–FF air mixing flow).

 5- The authors calculate the emission factor and advocate a cautious approach. However, the reader can only understand whether this magnitude is “reasonable” by comparing it with similar nanocomposite processing studies.

 Suggestion:

 Summarize the “machining/sanding/sawing” emission magnitudes (mass or count) in a table with at least 4–6 references.

 If mass emission is underreported, the uncertainty in the conversion from count emissions to “approximate mass” is debatable, as mentioned by the author; But at least a comparison of the order of magnitude should be given.

 6- In CoU calculations, the NF concentration target under RWC is selected as <0.5×OEL and <1×OEL and is associated with AIHA Exposure Category 2–3.

 This selection may be correct; however, a clearer answer to the question "why these thresholds?" is needed.

 Recommendation:

 Summarize the practical meaning of AIHA categories (control hierarchy / monitoring requirement) in one paragraph.

 It should be clearly stated that "RCR<1" is the standard in the context of REACH; "0.5×OEL" is selected as a conservative target for "highly controlled".

 7- The results state that the particles are concentrated in the inhalable fraction by weight and the GMD is ~10 µm (also in the abstract).

 This is important from an occupational hygiene perspective; however, some visual elements in the figures reduce readability (yellow bars) and a stronger description of the size distribution could be used.

 Suggestion:

 Add panel-based or at least separate PU-GB vs. PU-DIA average distributions for “dN/dlogDp” and “dm/dlogDp” from ELPI and OPC. Include a short reference set for inhalable/respirable distinction.

Is the study design appropriate and does the work have academic merit?

Yes

Is the work clearly and accurately presented and does it cite the current literature?

Partly

If applicable, is the statistical analysis and its interpretation appropriate?

Partly

Are all the source data underlying the results available to ensure full reproducibility?

Partly

Are the conclusions drawn adequately supported by the results?

Partly

Are sufficient details of methods and analysis provided to allow replication by others?

Partly

Reviewer Expertise:

Internal combustion engines; emission analysis and control; alternative and biofuels; experimental optimization and modeling of combustion and emissions.

I confirm that I have read this submission and believe that I have an appropriate level of expertise to confirm that it is of an acceptable scientific standard, however I have significant reservations, as outlined above.

# Reviewer report: Reviewer response for version 2

**DOI:** 10.21956/openreseurope.23006.r63240

**Published:** 2025-12-03

**Authors:** Ramazan Şener

**Affiliations:** 1 Bandırma Onyedi Eylül University, Balıkesir, Turkey

The manuscript addresses an important occupational health and environmental safety question related to particle emissions from cutting nano-enabled biobased polyurethane foams. The contribution is scientifically meaningful; however, several sections require clarification, deeper explanation, improved methodological transparency, and textual refinement to enhance reproducibility and scientific rigor:

1. The rationale behind using functionalized silica, GasBeton, and Diatomite nanofillers should be briefly explained in the introduction.
2. The term “RWC” must be defined earlier, including assumptions and justification.
3. Include references that distinguish inhalable vs respirable emissions, to contextualize the significance of 10 µm GMD.
4. This section needs more detail to ensure reproducibility. Provide specifications of functionalized silica, GasBeton, and Diatomite, also the exact loading percentages.
5. Cutting speed, blade type, temperature, humidity, and background ventilation parameters must be described explicitly.
6. Sampling instrumentation should be described with detection range and uncertainty quantification.
7. Please explain the rationale behind choosing 0.5xOEL and 1xOEL thresholds.
8. The finding that emissions are predominantly inhalable is important, but the manuscript should include more detailed size distribution plots.
9. The aggregated emission factor requires comparison with previous studies to contextualize magnitude.
10. The CoU results are interesting, but additional explanation is needed.
11. The discussion should be strengthened by comparing emission factors with literature on machining of nanocomposites and explaining mechanisms behind high inhalable particle dominance and addressing limitations.

Is the study design appropriate and does the work have academic merit?

Yes

Is the work clearly and accurately presented and does it cite the current literature?

Partly

If applicable, is the statistical analysis and its interpretation appropriate?

Not applicable

Are all the source data underlying the results available to ensure full reproducibility?

Partly

Are the conclusions drawn adequately supported by the results?

Partly

Are sufficient details of methods and analysis provided to allow replication by others?

Partly

Reviewer Expertise:

Computational fluid dynamics; Particle emissions; combustion

I confirm that I have read this submission and believe that I have an appropriate level of expertise to confirm that it is of an acceptable scientific standard, however I have significant reservations, as outlined above.

# Response

**Published:** 2025-12-19

**Authors:** Antti Joonas Koivisto

We are grateful for the valuable comments. Unfortunately, we could not respond to all the comments due to missing information.   The manuscript addresses an important occupational health and environmental safety question related to particle emissions from cutting nano-enabled biobased polyurethane foams. The contribution is scientifically meaningful; however, several sections require clarification, deeper explanation, improved methodological transparency, and textual refinement to enhance reproducibility and scientific rigor:

1. The rationale behind using functionalized silica, GasBeton, and Diatomite nanofillers should be briefly explained in the introduction.

We added the description to the “Introduction” section.

1. The term “RWC” must be defined earlier, including assumptions and justification.

We added “i.e., conditions that can be achieved and lead to the highest emissions”

1. Include references that distinguish inhalable vs respirable emissions, to contextualize the significance of 10 µm GMD.

We added “ *Based on geometric mass mean diameter (Table 2), the released particles were mainly inhalable particles (Schwarz et al., 2018* *[JK1]* *).*”

1. This section needs more detail to ensure reproducibility. Provide specifications of functionalized silica, GasBeton, and Diatomite, also the exact loading percentages.

We clarified that the concentrations are in wt.%. We added “ *GasBeton ^®^ and diatomite were commercially available fillers (Extended Data;* *Koivisto et al., 2025d* *), and functionalized silica nanoparticles were derived from rice husks as described by* *Bengalli et al. (2025* *).*”

1. Cutting speed, blade type, temperature, humidity, and background ventilation parameters must be described explicitly.

We added “ *Cutting blade (Blade Widia) dimensions were* *34 mm (depth) x 4120 mm (length) x 2 mm (thickness) and the blade speed was 150 m/min.*“ Unfortunately, we don’t have relative humidity measurements or for the ventilation, other than “ *The cutting room is naturally ventilated via an entrance mesh door*” and “ *The random airflow velocity at the mesh door was 0.17±0.08 m/s (±STD)*.” However, the particle loss rate accounts for both particle deposition and removal by ventilation, which is essential for emission rate calculations.  

1. Sampling instrumentation should be described with detection range and uncertainty quantification.

The particle size detection range is given. Uncertainty quantification requires size-resolved mass concentration in the cutting room during cutting operations. This type of calibration procedure is not feasible to conduct within the project. Thus, we can only use the instrument's responses and the inversion routines it employs. No changes were made.  

1. Please explain the rationale behind choosing 0.5xOEL and 1xOEL thresholds.

Thresholds are based on AIHA exposure categories 2 and 3, which specify the need for exposure controls. This approach is more convenient than ECHA approach, which considers only controlled/uncontrolled exposure and allows 10% overexposure. ECHA   This is explained in the section “Conditions of Use” as “ *This corresponds to the American Industrial Hygiene Association (AIHA) exposure category 2 (highly controlled exposure), and **chemical-specific hazard communication is required action** ( Hewett et al., 2006;* *Torres et al., 2014* *).*” and “ *The exposure is considered controlled (AIHA exposure category 3), **but in addition to chemical-specific hazard communication, exposure surveillance, medical surveillance, and work practice evaluation are also necessary**.*” We added in Introduction “ *This approach is considered more convenient than under REACH legislation, where exposure is considered adequately controlled if the exposure distribution 90th percentile (P90) is below the limit value, i.e., P90 < OEL (* *ECHA, 2016* *), which allows 10% overexposure and is a holistic approach, i.e., it considers whether actions are needed without a transition in control actions (Koivisto et al., 2025b).*” Koivisto et al., 2025b: Koivisto, A.J., Eliat, M., Jayjock, M., Hussein, T., Nicosia, A., 2025b. Quantifying relevant exposure determinants and conditions of use for welding emissions. Open Res Europe 5, 38. https://doi.org/10.12688/openreseurope.19498.3

1. The finding that emissions are predominantly inhalable is important, but the manuscript should include more detailed size distribution plots.

Detailed size distribution plots are given for background aerosol and both cutting processes in the Extended Data. No changes were made.

1. The aggregated emission factor requires comparison with previous studies to contextualize magnitude.

Unfortunately, we were unable to find prior studies on the mass emission factors (mass emissions) of PU cutting procedures. No changes were made.

1. The CoU results are interesting, but additional explanation is needed.

We added to the Introduction “ *CoU defines the operational conditions and risk management measures that result in adequately controlled exposure (see Koivisto et al. 2025b).*” Koivisto et al., 2025b: Koivisto, A.J., Eliat, M., Jayjock, M., Hussein, T., Nicosia, A., 2025b. Quantifying relevant exposure determinants and conditions of use for welding emissions. Open Res Europe 5, 38. https://doi.org/10.12688/openreseurope.19498.3  

1. The discussion should be strengthened by comparing emission factors with literature on machining of nanocomposites and explaining mechanisms behind high inhalable particle dominance and addressing limitations.

Unfortunately, there is very limited amount of studies considering mass emissions from mechanical treatment processes. We have revised the mechanical treatment emissions in 2017, which was updated in 2025 (Koivisto et al. 2017; 2025a). No changes were made. Koivisto et al. 2017: Koivisto AJ, Jensen ACØ, Kling KI, et al.: Quantitative material releases from products and articles containing manufactured nanomaterials: towards a release library. NanoImpact. 2017;5:119–132. 10.1016/j.impact.2017.02.001   Koivisto et al. 2025a: Koivisto AJ, Jayjock M, Hussein T, et al.: Particle emissions by mechanical treatment of nanocomposites: emission library and applications in exposure and risk assessment [version 3; peer review: 2 approved, 1 approved with reservations]. Open Res Europe. 2025a;5:63. 10.12688/openreseurope.19658.1

  [JK1]Schwarz, K., Pappa, G., Miertsch, H., Scheel, J., Koch, W., 2018. A methodology for the assessment of inhalation exposure to aluminium from antiperspirant sprays. Arch Toxicol 92, 1383–1392. https://doi.org/10.1007/s00204-017-2151-2

# Reviewer report: Reviewer response for version 2

**DOI:** 10.21956/openreseurope.23006.r62878

**Published:** 2025-11-22

**Authors:** Yuji Fujitani

**Affiliations:** 1 National Institute for Environmental Studies, Tsukuba, Japan

General comments:

 The reviewer appreciates that the authors have revised the manuscript according to most of the reviewers’ comments, which helped to clarify the study. However, some of the points raised in the previous review appear to have been only partially addressed. For the points that have not been revised, the reviewer would appreciate if the authors could provide clear explanations for why no changes were made. The following points remain unchanged from the initial draft. The page and line numbers refer to those in the original version of the manuscript.

 Minor comments:

 Page 4 left column, first paragraph

 The section reports particle number emissions and exposure levels from previous studies. It would be helpful to indicate the particle size range of these emissions, if available.

 Page 4 right column, second paragraph

 The last sentence appears somewhat repetitive with the earlier statements in this paragraph about the lack of measurement standards. You may consider merging or rephrasing to avoid redundancy.

 Page 6 right column, second paragraph

 Please provide the exact times of cleaning and preparation activities, as they appear to influence NF concentrations.

 Figures 2 and 3

 The yellow horizontal bars are hard to see and should be changed in color. Also, it would be better to draw the horizontal bar outside the frame of the figure so that it does not interfere with the data.

 Page 8 second and third paragraphs

 The comparison between gravimetric measurements and estimates from ELPI and OPC-NF is difficult to interpret, because the sampling periods, averaging times, collection/ measurement size ranges, and detection techniques differ. It seems that the main point is to note that the use of unit density in mass calculations should produce a systematic error, which was not observed here. Consider clarifying the purpose of this comparison for the reader.

 Page 8 fourth paragraphs

 Two loss rate values are reported for both NF and FF, but it is unclear why there are two values. Clarifying this in the text would help readers understand the results without referring to the Extended Data figures.

 “The previous comment suggests that, for example, the loss rates should be reported as follows: 15.9 and 13.2 1/h at NF for the lunch and afternoon periods, respectively.”

 Page 9 right column, second paragraph

 The measured random air velocity and resulting β value are reported, but the calculation method is not shown. Providing the formula or a brief description of how β was derived from the air velocity and cube geometry would help readers understand the result.

 Table 3a

 What is “G” means?

 “The reviewer has confirmed the description of G in the revised manuscript; however, it would be helpful to also provide a brief explanation in the caption of Table 3a, as well as for β.”

Is the study design appropriate and does the work have academic merit?

Yes

Is the work clearly and accurately presented and does it cite the current literature?

Partly

If applicable, is the statistical analysis and its interpretation appropriate?

Not applicable

Are all the source data underlying the results available to ensure full reproducibility?

No

Are the conclusions drawn adequately supported by the results?

Yes

Are sufficient details of methods and analysis provided to allow replication by others?

Partly

Reviewer Expertise:

My expertise is in aerosol measurement and inhalation exposure assessment. I can evaluate the experimental design, concentration measurements, and emission/exposure assessment.

I confirm that I have read this submission and believe that I have an appropriate level of expertise to confirm that it is of an acceptable scientific standard, however I have significant reservations, as outlined above.

# Response

**Published:** 2025-12-19

**Authors:** Antti Joonas Koivisto

We are grateful for the constructive and valuable comments. We hope the manuscript quality meets the requirements for accepting the publication.

 General comments:

 The reviewer appreciates that the authors have revised the manuscript according to most of the reviewers’ comments, which helped to clarify the study. However, some of the points raised in the previous review appear to have been only partially addressed. For the points that have not been revised, the reviewer would appreciate if the authors could provide clear explanations for why no changes were made. The following points remain unchanged from the initial draft. The page and line numbers refer to those in the original version of the manuscript.

 Minor comments:

 Page 4 left column, first paragraph

 The section reports particle number emissions and exposure levels from previous studies. It would be helpful to indicate the particle size range of these emissions, if available. We added “ *Particle size number distributions had geometric mean diameters below 100 nm, and the released fragments were several micrometers in size, as observed in electron microscope images. Free nanoparticles were not observed.*”

 Page 4 right column, second paragraph

 The last sentence appears somewhat repetitive with the earlier statements in this paragraph about the lack of measurement standards. You may consider merging or rephrasing to avoid redundancy. The second paragraph emphasizes the importance of emission measurements for the REACH regulation and CoU assessment. The third paragraph states that ECHA is not complying with the REACH requirements, but allows the use of qualitative exposure models that do not require information on emissions. This is one reason there are no particle-emission measurement standards, even though there are well-established methods to standardize particle emission measurements. No changes were made.

 Page 6 right column, second paragraph

 Please provide the exact times of cleaning and preparation activities, as they appear to influence NF concentrations. Unfortunately, we do not have preparation or vacuum cleaning times. The main purpose of vacuum cleaning after cutting was to collect dust for dustiness tests. Other cleaning methods were used to ensure that initial settled dust levels were similar for both GasBenton and Diatomite cuttings. No changes were made.

 Figures 2 and 3

 The yellow horizontal bars are hard to see and should be changed in color. Also, it would be better to draw the horizontal bar outside the frame of the figure so that it does not interfere with the data. We are sorry, but we would not like to make changes to Figures 2 and 3. We have given the times in the main text, and the yellow bar cannot be mixed with the measurement data due to non-physical structure (straight line). This is just due to workload (change the figure, export, upload the figure, change the figures to the paper). We appreciate it if the current Figures can be used.

 Page 8 second and third paragraphs

 The comparison between gravimetric measurements and estimates from ELPI and OPC-NF is difficult to interpret, because the sampling periods, averaging times, collection/ measurement size ranges, and detection techniques differ. It seems that the main point is to note that the use of unit density in mass calculations should produce a systematic error, which was not observed here. Consider clarifying the purpose of this comparison for the reader. We added *“...because the sampling periods, averaging times, collection/ measurement size ranges, and detection techniques differ.*”

 Page 8 fourth paragraphs

 Two loss rate values are reported for both NF and FF, but it is unclear why there are two values. Clarifying this in the text would help readers understand the results without referring to the Extended Data figures.

 “The previous comment suggests that, for example, the loss rates should be reported as follows: 15.9 and 13.2 1/h at NF for the lunch and afternoon periods, respectively.” We added “ *The average loss rate was applied for the calculations because the conditions for the loss rate should have remained the same for both NF and FF. A loss rate assessment based on two measurements in NF and FF, respectively, is insufficient for uncertainty assessment.*” We consider the variation in loss rates in NF and FF due to assessment uncertainty, which cannot be evaluated from these measurements. We do not have a justification to change the loss rates during the day or to use the average loss rate. We used the average loss rate to simplify modelling and avoid over-credibility, considering the modelling quality. 

 Page 9 right column, second paragraph

 The measured random air velocity and resulting β value are reported, but the calculation method is not shown. Providing the formula or a brief description of how β was derived from the air velocity and cube geometry would help readers understand the result.

   Beta is calculated from the FF steady state equation (V _FF_ × d *C _OPC-FF_*/d *t* = 0). We added the equations to “Aerosol dynamic model” section the solutions: “ Eq1.                                  (1)   Eq2.                                   (2) *Where C _OPC-NF_ and C _OPC-FF_ are measured average concentrations (µg/m ^3^) at NF and FF, respectively, and Q is the product of room volume (171 m ^3^) and particle loss rate (1/min). All particles are assumed to be removed from the FF volume, and the NF volume is open-faced (not in contact with walls or screens).*”

 Table 3a

 What is “G” means?

 “The reviewer has confirmed the description of G in the revised manuscript; however, it would be helpful to also provide a brief explanation in the caption of Table 3a, as well as for β.” These were added to Table 3a.

# Reviewer report: Reviewer response for version 1

**DOI:** 10.21956/openreseurope.22511.r57944

**Published:** 2025-08-23

**Authors:** Yuji Fujitani

**Affiliations:** 1 National Institute for Environmental Studies, Tsukuba, Japan

General comments:

 The manuscript investigates particle emissions generated during the cutting of cured bio-based polyurethane foams containing nanofillers. Emissions were quantified based on near- and far-field measurements, corrected for background concentrations, and translated into emission factors to support conditions-of-use assessments under REACH. Both manual and automated cutting scenarios were considered, and the resulting exposure levels were classified as highly controlled or controlled. The work presents a method for assessing emissions under real working conditions and defines broadly applicable conditions of use in compliance with REACH requirements. Overall, the manuscript is clearly structured; however, it could benefit from minor English language editing to further improve readability.

 Minor comments:

 Introduction

 The introduction provides extensive background on PU materials, nanofillers, and regulatory requirements; however, it does not clearly highlight the novelty or key contribution of the present study. The gap in knowledge regarding mass-based emission data for cutting PU panels should be explicitly stated, and the specific aims and significance of this study should be emphasized. I recommend revising the introduction to clearly convey what is new and important about this work, so that readers can immediately understand its relevance and impact.

 Page 4 left column, first paragraph

 The section reports particle number emissions and exposure levels from previous studies. It would be helpful to indicate the particle size range of these emissions, if available.

 Page 4 right column, first paragraph

 In this section, the term “emission assessment” is used. Since REACH Annex I §5.2.1 specifies “emission estimation,” it may be clearer to align the terminology with the regulation.

 Page 4 right column, second paragraph

 The last sentence appears somewhat repetitive with the earlier statements in this paragraph about the lack of measurement standards. You may consider merging or rephrasing to avoid redundancy.

 Page 4 right column, third paragraph

 The term “cutting intensity” is unclear. It seems to refer to the amount or rate of cutting (e.g., area cut per unit time or number of panels), but a more precise definition would help the reader.

 Page 5 Instrumentation

 It is unclear whether tubing was used for the sampler or instrument inlets. Please clarify, as this can affect measurement reliability.

 Page 6 left column, first and second paragraphs

 It would be helpful to clarify that “volume” refers to air volume, and to provide the units for the NF–FF air mixing rate (β), as this would improve understanding of the model.

 Page 6 right column, first paragraph

 The direction of the airflow velocity is not specified. Providing information on airflow direction would help clarify particle transport and exposure assessment.

 Page 6 right column, second paragraph

 Please provide the exact times of cleaning and preparation activities, as they appear to influence NF concentrations.

 Page 6 right column, second paragraph

 The expression “within a factor of ~2 similar” for particle size distributions is unclear. Please consider rephrasing.

 Figures 2 and 3

 The yellow horizontal bars are hard to see and should be changed in color. Also, it would be better to draw the horizontal bar outside the frame of the figure so that it does not interfere with the data.

 Table 2

 OPC-FF GMD value after subtracted background data seems too large. Please check the value to make sure it is really correct.

 Page 8 second and third paragraphs

 The comparison between gravimetric measurements and estimates from ELPI and OPC-NF is difficult to interpret, because the sampling periods, averaging times, collection/ measurement size ranges, and detection techniques differ. It seems that the main point is to note that the use of unit density in mass calculations should produce a systematic error, which was not observed here. Consider clarifying the purpose of this comparison for the reader.

 Page 8 fourth paragraphs

 Two loss rate values are reported for both NF and FF, but it is unclear why there are two values. Clarifying this in the text would help readers understand the results without referring to the Extended Data figures.

 Page 9 left column, first paragraph

 The NF volume is reported as 8 m³, but the rationale for this choice is not provided. A range of 4–12 m³ is also presented in Table 3b, yet it is unclear how this range was determined. Clarifying the basis for these NF volume values would improve transparency.

 Page 9 right column, second paragraph

 The measured random air velocity and resulting β value are reported, but the calculation method is not shown. Providing the formula or a brief description of how β was derived from the air velocity and cube geometry would help readers understand the result.

 Table 3a

 What is “G” means?

 Table 3b

 The text states that the range of loss rates is adopted from Table 2, but the values appear to be in Table 3a. Please check and correct the table reference if necessary.

 Page 10 left column, third paragraph

 Please rephrase“Table 3 parameters”as“parameters in Table 3”, which is clearer.

 Page 10, left column (fourth paragraph) through right column (first paragraph)

 This paragraph is difficult to read due to the large number of numerical results presented in one place. For example, in the section discussing NF and FF results, the sentence “The simulated concentrations overestimated the measured concentrations by a factor of 1.18” is confusing. It immediately follows the description of the FF results, but the factor of 1.18 actually corresponds to the NF comparison (23.4 vs. 19.8 μg/m³). Please clarify this explicitly, as otherwise it could be misinterpreted as applying to the FF results as well. I recommend restructuring the paragraph to improve readability.

 Page 11 right column, third paragraph

 Please spell out “NPs” (nanoparticles) when it first appears.

Is the study design appropriate and does the work have academic merit?

Yes

Is the work clearly and accurately presented and does it cite the current literature?

Partly

If applicable, is the statistical analysis and its interpretation appropriate?

Not applicable

Are all the source data underlying the results available to ensure full reproducibility?

No

Are the conclusions drawn adequately supported by the results?

Yes

Are sufficient details of methods and analysis provided to allow replication by others?

Partly

Reviewer Expertise:

My expertise is in aerosol measurement and inhalation exposure assessment. I can evaluate the experimental design, concentration measurements, and emission/exposure assessment.

I confirm that I have read this submission and believe that I have an appropriate level of expertise to confirm that it is of an acceptable scientific standard, however I have significant reservations, as outlined above.

# Response

**Published:** 2025-10-20

**Authors:** Antti Joonas Koivisto

**General comments:**

 The manuscript investigates particle emissions generated during the cutting of cured bio-based polyurethane foams containing nanofillers. Emissions were quantified based on near- and far-field measurements, corrected for background concentrations, and translated into emission factors to support conditions-of-use assessments under REACH. Both manual and automated cutting scenarios were considered, and the resulting exposure levels were classified as highly controlled or controlled. The work presents a method for assessing emissions under real working conditions and defines broadly applicable conditions of use in compliance with REACH requirements. Overall, the manuscript is clearly structured; however, it could benefit from minor English language editing to further improve readability.   We thank the reviewer for the extensive revision and highly valuable comments. The review has significantly increased the study quality. We hope the specific comments are addressed adequately.

**Minor comments:**

**Introduction**

 The introduction provides extensive background on PU materials, nanofillers, and regulatory requirements; however, it does not clearly highlight the novelty or key contribution of the present study. The gap in knowledge regarding mass-based emission data for cutting PU panels should be explicitly stated, and the specific aims and significance of this study should be emphasized. I recommend revising the introduction to clearly convey what is new and important about this work, so that readers can immediately understand its relevance and impact.  

**Response: **The novelty of this study lies in the assessment of emission factors for this specific PU panel cutting. Methods and measurement techniques have been in existence for decades. Their application for the REACH regulation is possible but unlikely because the European Chemicals Agency (ECHA) allows the use of qualitative models in quantitative exposure assessment under REACH (Koivisto and Jayjock, 2025; https://doi.org/10.12688/openreseurope.19510.2). This creates a conflict that is challenging to address in this article and may cause confusion, which we would like to avoid here. We aim to present evidence that a quantitative CoU assessment, in compliance with the REACH regulation, is feasible.   We rephrased the last sentence as: “Overall, this study demonstrates how to conduct quantitative emissions analyses using mass balance principles, how to calculate emission factors for cutting, and how to use the emission factors to quantify CoU that comply with the REACH regulation.”   The reference was corrected in the Introduction's third paragraph.

 Page 4 left column, first paragraph

 The section reports particle number emissions and exposure levels from previous studies. It would be helpful to indicate the particle size range of these emissions, if available.  

**Response: ** Neubauer et al. (2017) do not report particle size. Ding et al. (2017) used DISCmini to measure geometric mean diameter for thermoplastic polyurethane composites during drilling. Geometric mean particle diameter ranged from 45 (TPU containing SiO2 NPs) to 220 nm (TPU containing carbon nanotubes) NPs for particle number concentrations (mass concentrations were not reported), depending on the filler type. In DISCmini, the diameter measurement is based on the concentration differences between the diffusion and filter stages for aerosol, where large particles are removed using a cyclone with a cut size of 700 nm. The diameter is challenging to compare with our measurements due to different materials, measurement techniques, and processes. In our study, the particle GMD was on average 20 nm based on ELPI measurement (Table 2). No changes were made.

 Page 4 right column, first paragraph

 In this section, the term “emission assessment” is used. Since REACH Annex I §5.2.1 specifies “emission estimation,” it may be clearer to align the terminology with the regulation. **Response: **This was corrected.

 Page 4 right column, second paragraph

 The last sentence appears somewhat repetitive with the earlier statements in this paragraph about the lack of measurement standards. You may consider merging or rephrasing to avoid redundancy.

**Response: **The last sentence is referring to emission models, while the previous section refers to measurement standards. It means that there are no particle measurement standards nor particle emission models. No changes were made.

 Page 4 right column, third paragraph

 The term “cutting intensity” is unclear. It seems to refer to the amount or rate of cutting (e.g., area cut per unit time or number of panels), but a more precise definition would help the reader.

**Response: **This was changed to cutting rate (m ^2^/min).

 Page 5 Instrumentation

 It is unclear whether tubing was used for the sampler or instrument inlets. Please clarify, as this can affect measurement reliability.

**Response: **The ELPI, OPC-NF, and PM sampler inlets were placed at a height of 150 cm and between the band saw blade and the operator. **Sampling was performed for ELPI, OPC-FF and PM samplers using conductive sampling tubes with an inner diameter of 8 mm and a length of 205 cm for ELPI and 130 cm for OPC-FF and PM samplers.** **Diffusion losses by the sampling lines were not corrected because the contribution is negligible to the mass concentrations, and the loss correction cannot be applied to the PM samplers.** The OPC-FF inlet was ca. 7 m from the NF site at a height of 100 cm **(without sampling line)**.

 Page 6 left column, first and second paragraphs

 It would be helpful to clarify that “volume” refers to air volume, and to provide the units for the NF–FF air mixing rate (β), as this would improve understanding of the model.

**Response: **These were added to both paragraphs.

 Page 6 right column, first paragraph

 The direction of the airflow velocity is not specified. Providing information on airflow direction would help clarify particle transport and exposure assessment.

**Response: **This was a single-wire anemometer, and the flow direction cannot be measured. We clarified this in methodology as “Random airflow measurements were conducted using a single hot-wire anemometer (Testo Inc., model Testo 405i, Pennsylvania, US) with a detection limit of 0.01 m/s.” and in the Results section that this was a random air velocity.

 Page 6 right column, second paragraph

 Please provide the exact times of cleaning and preparation activities, as they appear to influence NF concentrations.

**Response: **We modified the sentence as “Before cutting, **panels were moved to the room, instruments were set up, and residual dusts from the previous cutting operations were wiped from the band saw**, increasing the NF concentrations (Figure 2 and Figure 3).”

 Page 6 right column, second paragraph

 The expression “within a factor of ~2 similar” for particle size distributions is unclear. Please consider rephrasing.

**Response: **We clarified this by adding references (bolded) “The background concentration ratios between the NF and FF OPCs were 1.3 and 0.9 for particle number and mass concentrations **(Table 1)**, respectively, and the background size distributions were within a factor of ~2 similar in the NF and FF **(Figures ED1 and ED2, Extended Data; (** **Koivisto *et al*., 2025d** **))**.”

 Figures 2 and 3

 The yellow horizontal bars are hard to see and should be changed in color. Also, it would be better to draw the horizontal bar outside the frame of the figure so that it does not interfere with the data.

**Response: **We agree on Figure 2, but we would like to keep the same color as in Figure 3 and also associate well the period with the concentrations. We would like to keep the original color, because reader can zoom into the document and see better the measurement period compared to the concentrations measured by the ELPI and NF/FF OPCs.

 Table 2

 OPC-FF GMD value after subtracted background data seems too large. Please check the value to make sure it is really correct.

**Response: **Table values are misleadingly given in nm and not µm as they should be. The values were divided by 1000 to correct the units.

 Page 8 second and third paragraphs

 The comparison between gravimetric measurements and estimates from ELPI and OPC-NF is difficult to interpret, because the sampling periods, averaging times, collection/ measurement size ranges, and detection techniques differ. It seems that the main point is to note that the use of unit density in mass calculations should produce a systematic error, which was not observed here. Consider clarifying the purpose of this comparison for the reader.

**Response: **The second paragraph compares the background concentrations measured with the process concentrations measured by the online instruments. Aerosol characteristics during the background measurements and process measurements vary, including the effective density, which results in a systematic error. In this case, measurement techniques do not cause error because they remain the same, and only the assumption regarding the effective density causes an error. We added “Background particle density is expected to differ from process particle density, and the uncertainty in this difference is not known.” The third paragraph systematic error is related to all measurement differences mentioned above. This is discussed in the third paragraph.

 Page 8 fourth paragraphs

 Two loss rate values are reported for both NF and FF, but it is unclear why there are two values. Clarifying this in the text would help readers understand the results without referring to the Extended Data figures.

**Response: **This was clarified by adding the following sentence: “Coarse particles may settle before reaching the FF measurement location. To obtain a representative loss rate, loss rates were calculated from both NF and FF concentrations to determine if there were any significant differences.“

 Page 9 left column, first paragraph

 The NF volume is reported as 8 m³, but the rationale for this choice is not provided. A range of 4–12 m³ is also presented in Table 3b, yet it is unclear how this range was determined. Clarifying the basis for these NF volume values would improve transparency.

**Response: **We added the following description to Table 3b: “This represents a cube with a side length ranging from 1.6 to 2.3 m, which mimics the operator working distance for cutting (0.8 m) and replacing the panel with a new one (1.2 m).”

 Page 9 right column, second paragraph

 The measured random air velocity and resulting β value are reported, but the calculation method is not shown. Providing the formula or a brief description of how β was derived from the air velocity and cube geometry would help readers understand the result.

**Response: **This is referred to in the Aerosol dynamic model section, second paragraph, as “Air mixing between the NF and FF ( *β*) and an initial estimate for the emission rate were calculated using the NF and FF steady state concentrations and the particle loss rate by general ventilation and deposition (TEAS model 2Box.CE.Gv.SS; Ganser & Hewett, 2017).” We encourage looking at the equations and their derivation from the original study. This is independent of the NF geometry. Emission rate (G, mg/min) is a product of loss rate (ventilation and deposition) and  FF concentration [G (mg/min) = C_FF (mg/m3) * Q (m3/min)]. Beta (m3/min) is a product of generation rate and the concentration difference between NF and FF [beta (m3/min) = G (mg/min)/(C_NF (mg/m3) – C_FF (mg/m3))].

 Table 3a

 What is “G” means?

**Response: **We added in the Aerosol dynamic model section the explanation as: “Air mixing between the NF and FF ( *β*, m ^3^/min) and an initial estimate for the emission rate ( ***G*, mg/min**) were calculated using the NF and FF air volumes steady state concentrations and the particle loss rate by general ventilation and deposition (TEAS model 2Box.CE.Gv.SS; Ganser & Hewett, 2017).”

 Table 3b

 The text states that the range of loss rates is adopted from Table 2, but the values appear to be in Table 3a. Please check and correct the table reference if necessary.

**Response: **This was corrected.

 Page 10 left column, third paragraph

 Please rephrase“Table 3 parameters”as“parameters in Table 3”, which is clearer.

**Response: **This was clarified.

 Page 10, left column (fourth paragraph) through right column (first paragraph)

 This paragraph is difficult to read due to the large number of numerical results presented in one place. For example, in the section discussing NF and FF results, the sentence “The simulated concentrations overestimated the measured concentrations by a factor of 1.18” is confusing. It immediately follows the description of the FF results, but the factor of 1.18 actually corresponds to the NF comparison (23.4 vs. 19.8 μg/m³). Please clarify this explicitly, as otherwise it could be misinterpreted as applying to the FF results as well. I recommend restructuring the paragraph to improve readability.

**Response: **The comparison of measured OPC-FF and simulated FF concentrations was moved to the end, and the text was clarified.

 Page 11 right column, third paragraph

 Please spell out “NPs” (nanoparticles) when it first appears.

**Response: **This was spelled out and added to the abbreviations list.
